# Histological investigation of the interthalamic adhesion and periventricular region: Evidence for midline neural connectivity

**DOI:** 10.1002/ibra.12200

**Published:** 2025-06-13

**Authors:** Nicole van Heerden, Lané Prigge, Gerda Venter

**Affiliations:** ^1^ Department of Anatomy School of Medicine, Faculty of Health Sciences, University of Pretoria Pretoria Gauteng South Africa

**Keywords:** diencephalon, histology, interthalamic adhesion, periventricular region, thalamus

## Abstract

The interthalamic adhesion (IA), which may not be present in all humans, is a midline structure that connects the two thalami within the brain's third ventricle. A review of the known literature regarding the IA shows few histological studies and controversy regarding the organization of neurons within this region. This study conducted an anatomical investigation of the human IA in adult South African samples. Samples were obtained from 20 human adult embalmed cadavers: 11 from brains with a visible IA and 9 from brains without this feature. All the samples were harvested using sagittal sections of the area. Three additional samples were sectioned horizontally, yielding 33 tissue blocks. Before observation, these samples were appropriately processed for light microscopy and stained with haematoxylin and eosin, as well as cresyl violet. The results showed that no specific structural arrangements of the neurons were identifiable. The appearance appeared random, except for a distinguishable range in the frequency and dispersion of specific cells upon basic observation. Microglia were the most abundant cell type, and blood vessels were also observed. This study reports a novel inspection of the general histology of the thalamus, specifically of the IA and the periventricular (PVR) region, in midsagittal sections and three horizontal sections. This study confirmed the presence of pyramidal neurons within the IA, forming a bridge between the PVR region of the thalami, thus providing evidence to suggest that the IA could serve as a potential bridge for neural connections crossing over the brain's midline.

## Graphical abstract

This study investigated the interthalamic adhesion histology in human adult South African cadavers, revealing random cytoarchitecture with abundant microglia and widespread neurons observed via Cresyl Violet staining. Findings suggest the interthalamic adhesion may serve as a neural bridge between thalami, offering insights into midline brain connectivity.

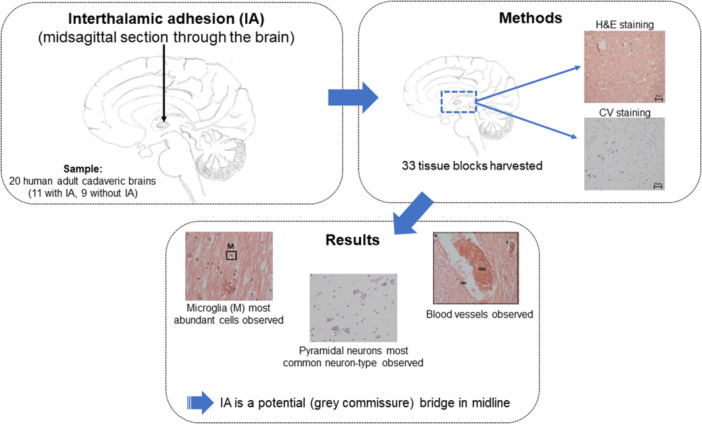

## 1 INTRODUCTION

The interthalamic adhesion (IA) is a midline, gray matter structure within the third ventricle that connects the two thalami of the brain.^1^, ^2^ Compared to other mammals, the human IA is much smaller and may be absent, is more variable in size, and consists of fewer nuclei.^3^ If present, it forms during the 13th and 14th weeks of gestation as a fusion of the medial boundaries of the two thalami.^4^

It has been established that available literature pertaining to the IA is relatively scarce; therefore, little information exists regarding its function within the brain. Only in the preceding decades has an increase in this subject been seen. In addition, there is a general lack of specialized brain tissue regions in general histological study samples. The general histological composition of the IA within known literature is not clearly described despite histological depictions of other structures in the brain, resulting in a void of information.^5^

There is controversy in the literature regarding the organization and function of neurons within the IA. Therefore, further research is necessary to reach a reliable consensus. To date, the most informative results were provided by Laslo and co‐authors.^6^ The authors reported that all six specimens in their sample, sliced into frontal/coronal sections, showed a circular arrangement of neurons in both the IA and periventricular (PVR) regions. These arrangements were formed by an average of 7.29 neurons, of which fusiform neurons numbered the most.^6^, ^7^ The authors concluded that the IA plays an active role in the brain, a finding supported by other studies in the existing literature.^6^, ^8^, ^9^ In contrast, the IA has been previously viewed and described as a vestigial structure in the brain.^10^

This study aimed to conduct an anatomical investigation of the human IA at a cellular level in an adult sample from the South African population, adding to the academic literature the information about its cellular anatomy and function within the brain and clinical significance.

## 2 MATERIALS AND METHODS

Sections were obtained from 21 embalmed human adult cadaveric brain specimens aged 56–95 years, donated to the Department of Anatomy at the University of Pretoria, South Africa. However, one brain sample had to be excluded due to damage during preparation. Thus, 20 cadaveric brains yielded 11 specimens with a visible IA and 9 specimens without. Tissue blocks, no more than 2 cm^3^, were dissected for histological preparation from each brain specimen. Samples with an IA included blocks of both the IA and PVR regions, while samples without an IA only included the PVR region. All the IA and PVR samples were harvested using sagittal sections of the area. Three additional samples (two with IA and one PVR) were sectioned horizontally. This formed 33 tissue blocks, as summarized in Table 1.

**Table 1:** Sample description for sections and tissue blocks used in this study.

Unique identifier(s)		Section description	IA present	Total number of tissue blocks obtained from brain specimens
1	B	1 PVR	Yes	2
	C	1 IA		
2		1 IA	Yes	1
3		1 PVR	No	1
4	A	1 PVR	No	2
	B	1 Horizontal		
5	A	1 PVR	Yes	2
	B	1 IA		
6	A	1 PVR	Yes	2
	B	1 IA		
7	A	1 PVR	Yes	3
	B	1 IA		
	C	1 Horizontal		
8		1 PVR	No	1
9		1 PVR	No	1
10	A	1 PVR	Yes	2
	B	1 IA		
11	A	1 PVR	Yes	2
	B	1 IA		
13		1 PVR	No	1
14		1 PVR	No	1
15		1 PVR	No	1
16	A	1 PVR	Yes	2
	B	1 IA		
17		1 PVR	No	1
18		1 PVR	No	1
19	A	1 PVR	Yes	2
	B	1 IA		
20	A	1 PVR	Yes	3
	B	1 IA		
	C	1 Horizontal		
21	A	1 PVR	Yes	2
	B	1 IA		

*Note*: IA refers to interthalamic adhesion, while PVR refers to the periventricular region. Number 12 was excluded.

The histological preparation of the sample included embedding, sectioning, and staining processes. Tissue blocks were trimmed, labeled by cadaver number and region, and processed through phosphate buffer rinses, ethanol dehydration, and xylene clearing. They were infiltrated with increasing concentrations of molten wax, embedded in moulds, and left to harden on a cold plate. Once embedded, blocks were sectioned into 5 μm slices using a microtome, and sections were placed on microscope slides after flattening in a warm water bath.

Haemotoxylin and eosin (H&E) staining was performed using a standard protocol involving sequential xylene, ethanol, and distilled water rinses. Slides were stained with haematoxylin, treated with Scott's buffer, briefly counterstained with eosin, and then dehydrated before a final xylene rinse. Each slide was mounted with Entellan and a coverslip, with care taken to eliminate any air bubbles. Cresyl violet (CV) staining followed a standard protocol, beginning with sequential xylene, ethanol, and distilled water rinses. Slides were stained with CV for 5 min, briefly dipped in water, and then differentiated in 95% ethanol. Further rinses followed this in 100% ethanol and xylene. Finally, slides were mounted with Entellan and coverslips, ensuring no bubbles remained. The slides were observed with a light microscope with a camera at 5×, 10×, and 20× magnification.

## 3 RESULTS

The expected histological observations were first based on the findings published by Laslo and co‐workers.^6^ In their study, the authors observed a circular organization of neurons in the human IA and PVR. However, these results could not be replicated in our study. Therefore, the objectives of our study were adapted to record all possible significant observations, especially potential differences between the regions of the IA and the PVR. Due to the long‐term fixation of the cadaveric material used to source the histological sample, notable damage to the sample tissue was observed. This did not alter the overall observation of the cells and their various morphological types for our purposes. Vacuolation could be observed within our sample specimens, primarily adjacent to vessels or neurons, but not within the neurons.

No specific structural arrangements of the neurons in the IA or PVR were identifiable. Indeed, the architecture appeared random except for a distinguishable range in the frequency and dispersion of specific cells upon simple observation. The H&E staining resulted in pink cytoplasm coloring of the cells, with the nuclei darkly stained blue/purple. The most distinguishable components observed included arteries, veins, red blood cells, microglial cells, and pyramidal cells (corresponding to neurons). The CV staining resulted in an almost clear/transparent representation of the extracellular matrix and darkly blue/purple‐stained rough endoplasmic reticulum, thus significantly improving the visualization of the Nissl substance, the nuclear membrane, and the nucleoli of the cells.

**Figure 1:**
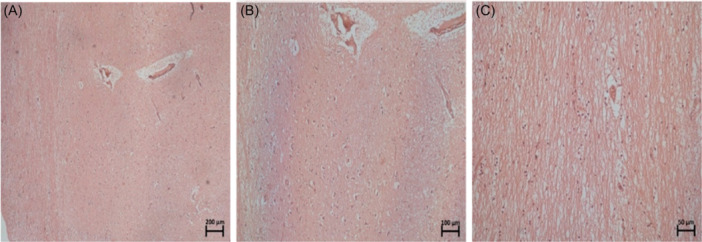
Specimen number 14 (PVR section without an IA) stained with H&E at 5× (A), 10× (B), and 20× (C) magnifications. H&E, haemotoxylin and eosin; IA, interthalamic adhesion; PVR, periventricular region. [Color figure can be viewed at wileyonlinelibrary.com]

The extracellular matrix staining is vastly different between the two stains, and H&E was more effective in visualizing any notable patterns in the cellular arrangement within the extracellular matrix. As seen in Figure 1, the sample without the IA appeared more striated towards the left edge than the smoother appearance towards the middle of the sample area.

An additional sample showed intermittent striations in the extracellular matrix. This observation is not attributed to chatter, as is sometimes seen in slide sections due to damage from the microtome while being sliced.

**Figure 2:**
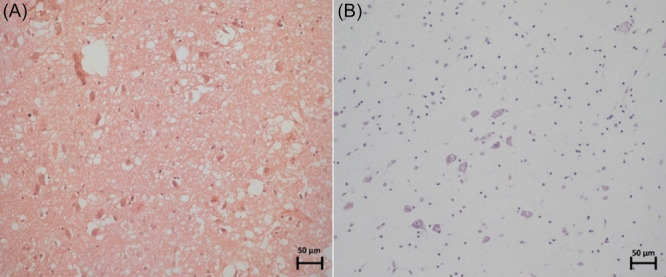
Specimen number 20 (Horizontal section through both IA and PVR) stained with (A) H&E compared to (B) CV stain, both at 20× magnification. CV, cresyl violet; H&E, haemotoxylin and eosin; IA, interthalamic adhesion; PVR, periventricular region. [Color figure can be viewed at wileyonlinelibrary.com]

Microglia were the most abundant cell type observed within the study sample. However, neurons were widespread throughout the specimens, which could only be observed with the CV staining. With careful observation, it is possible to slightly demarcate a few neuronal structures with the H&E staining (dark purple stains). Still, given the effort and high likelihood of failure, it is much better to rely on the CV staining for a more accurate observation of neuronal structures, as seen in Figure 2. The darker blue/purple stains indicate the neurons in this figure. The white spaces in the H&E stains are evidence of tissue vacuolation/damage. Another stain would have needed to be used to identify myelin, such as Luxol Fast Blue, but it was not used in this study. The thalamus is a known gray matter structure within the brain. CV staining was sufficient for the objectives of our study, which did not include identifying white matter within the IA and PVR regions. Therefore, we did not use the Luxol Fast Blue staining protocol.

**Figure 3:**
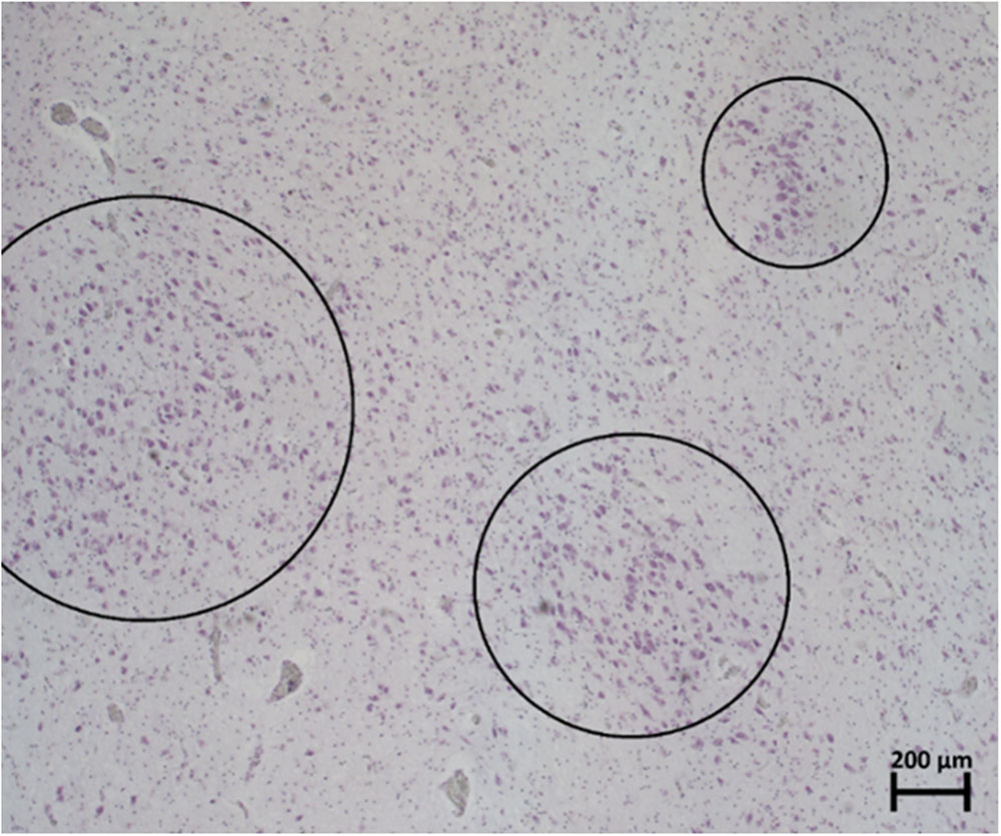
Specimen number 4 (PVR without IA) stained with CV‐staining at 10× magnification. The areas within the black circles indicate denser zones of neurons randomly dispersed through this periventricular region. CV, cresyl violet; IA, interthalamic adhesion; PVR, periventricular region. [Color figure can be viewed at wileyonlinelibrary.com]

From this study, there does not appear to be any pattern or structural organization in the appearance of neurons within the regions of interest, and zones of higher frequency of neurons dispersed randomly are seen in Figure 3. When viewed using light microscopy, neurons are typically characterized by their large cell bodies, Nissl substance (darkly stained with CV), and an evident nucleolus within their nucleus.^5^ In this sample, there appeared to be a monomorphic population of neurons (specifically pyramidal cells).

**Figure 4:**
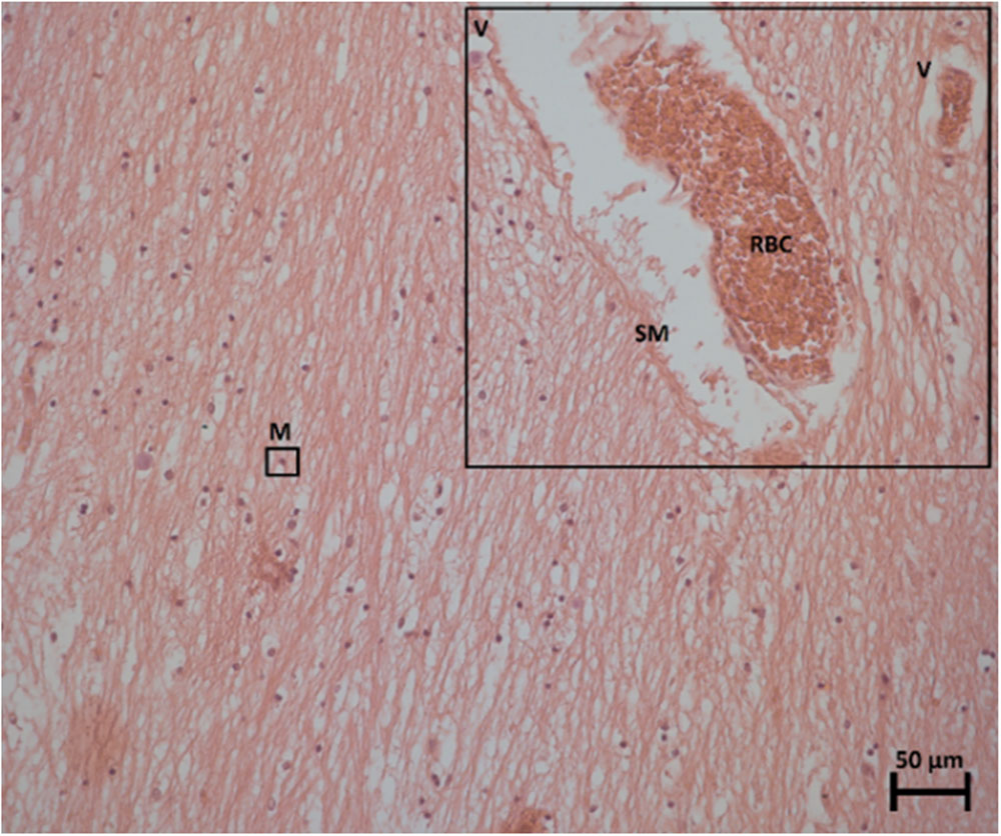
Specimen number 14 (PVR without IA) stained with H&E‐staining at 20× magnification depicting a larger and smaller vein (V) running alongside each other, where the thin, smooth muscle (SM) wall can be seen surrounding the red blood cells (RBC), and dark, purple‐stained microglial cells (M). IA, interthalamic adhesion; H&E, haemotoxylin and eosin; PVR, periventricular region. [Color figure can be viewed at wileyonlinelibrary.com]

Blood vessels were also observed in these areas of interest. The veins are identified by their collapsed appearance due to a thin or non‐existent smooth muscle wall known as the tunica media (Figure 4). A thicker wall of smooth muscle can distinguish an artery. Given that these specimens were cut in the sagittal plane, it can be deduced that these blood vessels run horizontally and laterally through the thalamus.

**Figure 5:**
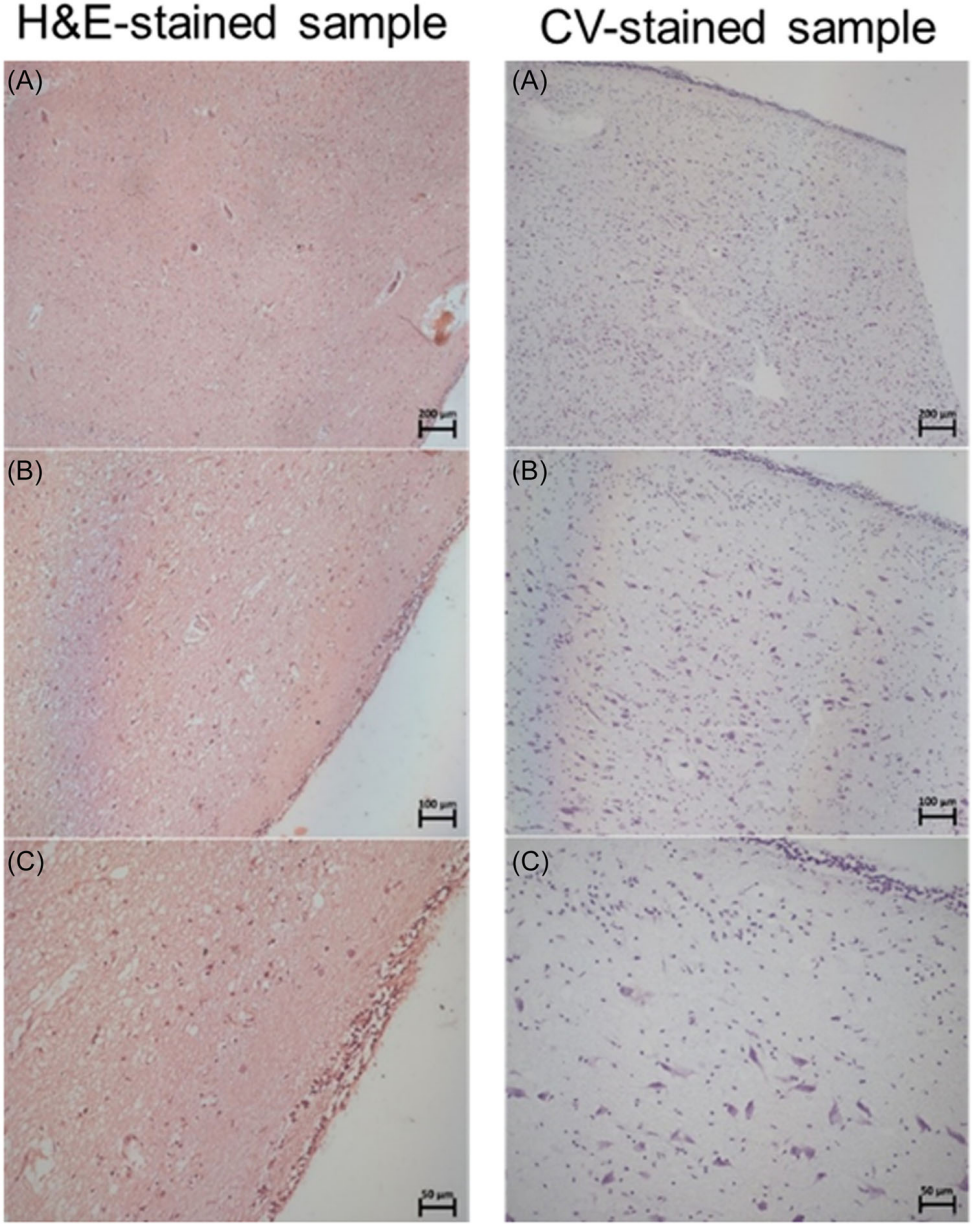
A comparison between the H&E‐stain and the CV‐stain of specimen number 14 (PVR without IA) at (A) 5× magnification, (B) 10× magnification, and (C) 20× magnification. CV, cresyl violet; H&E, haemotoxylin and eosin; IA, interthalamic adhesion; PVR, periventricular region. [Color figure can be viewed at wileyonlinelibrary.com]

In some of the histological sample images, a dense cellular layer of microglial cells on the edge of the tissue was observed. This was seen very clearly with the CV stain. In each instance, there appears to be an outer dense layer at the edge and an inner neuronal layer, with a sparse tissue layer of microglial cells between them (Figure 5).

**Figure 6:**
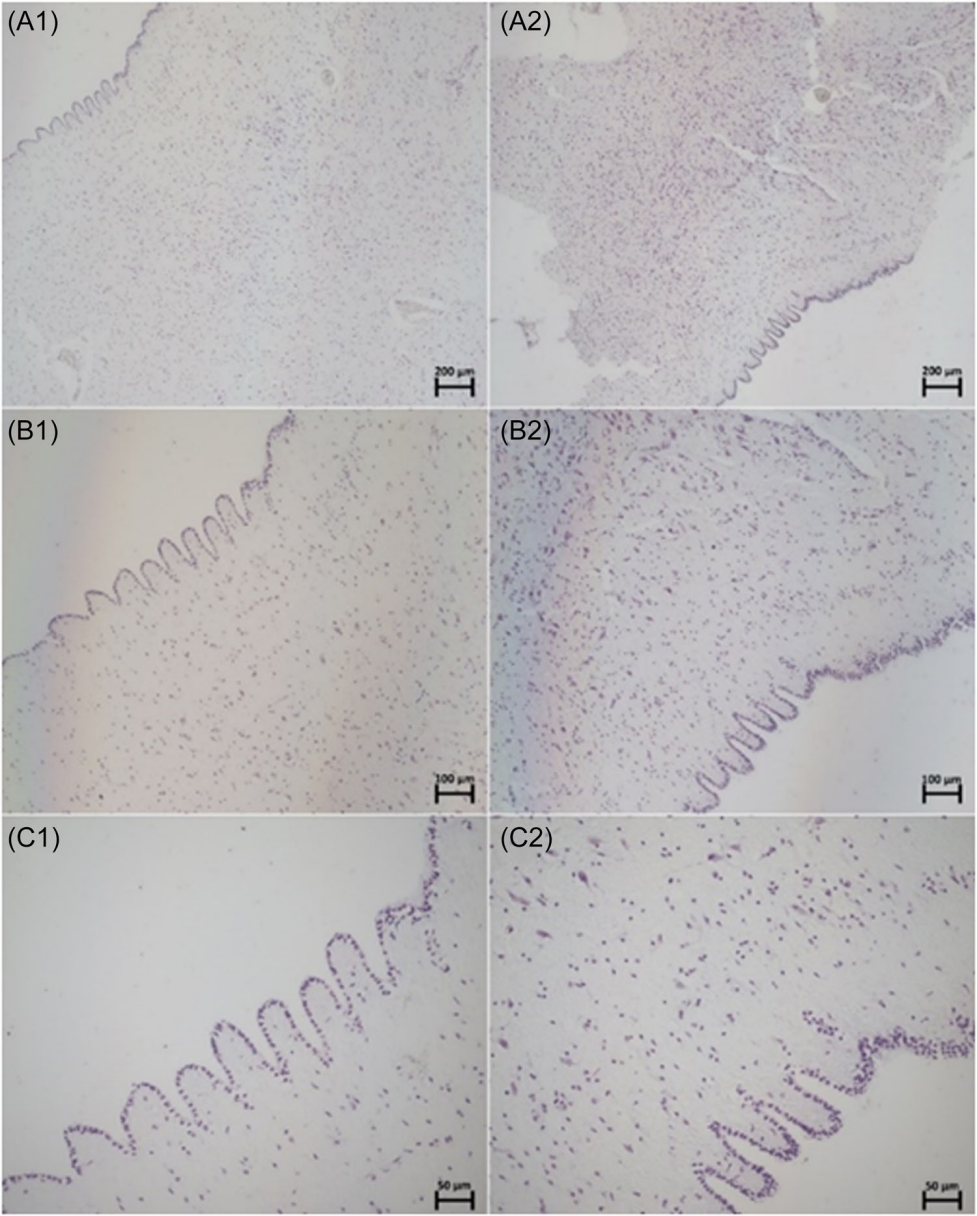
Specimen number 5 (PVR with IA) stained with CV at 5× (A), 10× (B), and 20× (C) magnifications at two regions. In the same sample, A1‐C1 appears to have a single‐layered lining, whereas area A2‐C2 appears to have 2–3 layers in the convoluted lining. CV, cresyl violet; IA, interthalamic adhesion; PVR, periventricular region. [Color figure can be viewed at wileyonlinelibrary.com]

Another sample showed a similar lining of densely packed cells. However, a section of this lining suddenly appears convoluted (Figure 6), possibly ascribed to the folding of the ependyma.

**Figure 7:**
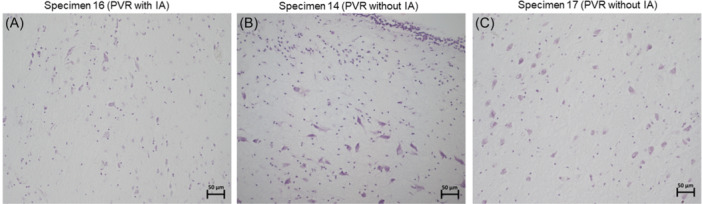
Comparison of CV‐stained specimens between PVR samples with an IA (A) and without an IA (B and C) at 20× magnification. CV, cresyl violet; IA, interthalamic adhesion; PVR, periventricular region. [Color figure can be viewed at wileyonlinelibrary.com]

Comparisons were conducted between the images of the samples with both the IA and PVR regions and between the PVR regions of the samples with an IA against the specimens without an IA (Figure 7). There appears to be a difference in glial cell density. However, these differences do not seem significant.

A comparison of the IA and PVR in the same sample with H&E staining reveals significantly more vacuolation and damage in the PVR region than in the IA region, together with the presence of a higher density of glial cells. A comparison of the IA and PVR in the same sample with CV staining also presented a higher density of glial cells in the IA region. The neuronal cells appear to have a similar orientation but no sophisticated structural organization.

When comparing the PVR in H&E‐stained specimens with and without an IA, blood vessels are common within this region. Although differences between the specimens with and without an IA do not appear significant, a notable difference is observed between certain specimens without an IA. Similarly, discrepancies in the density of glial cells were noted in the PVR of corresponding samples without an IA.

When comparing the PVR in CV‐stained specimens with and without an IA, there appears to be a more clearly observed difference in glial cell density than with H&E staining. Although differences between the specimens with and without an IA do not appear significant, there is again a notable difference between the two specimens without an IA. The PVR of specimens without an IA shows a much higher glial cell density than in specimens with an IA, in addition to the acute density of these cells that appear to form a cellular lining.

In specimens sectioned horizontally, as opposed to the sagittal sectioning of the rest of the sample, no significant differences were observable with H&E staining of glial and neuronal cell density and structural organization. Horizontal sections of the IA/PVR region appear less densely populated by microglial cells than the sagittal sections. This could mean that glial cell density may appear different depending on the orientation of the cellular structure and the level where the section is made.

## 4 DISCUSSION

The IA, also known as *massa intermedia*, is known for its highly variable prevalence, size, and shape.^11^, ^12^ Previously, this structure was reported to be a gray commissure containing cell bodies, but recent contradicting studies suggested it is a white commissure with no cell bodies.^11^

A review of the literature regarding the IA and PVR shows few histological studies in this region. Literature on general brain histology, specifically the thalamic region, is also scarce and might be ascribed to the known difficulty of stain‐impregnating the neurons in this area.^7^ This histological study was inspired by a study conducted and published by Laslo and co‐workers^6^ in 2005. Their study introduced the concept of a circular arrangement of neurons in the cytoarchitecture of the IA and PVR regions from their findings in a sample of six human brains with an IA. The methods of visualization of the histology of the cells utilized in this study differ from those used by Laslo and co‐authors.^6^ Different techniques utilized between the studies can be attributed to the extensive time between the two studies, in which histological methods have changed and improved, or are merely based on preferred histological observation methods. Our sample included both sagittal‐ and horizontal sections 5 μm thick and stained with H&E and CV dyes. In contrast, Laslo and co‐workers^6^ included only frontal sections 10 μm thick and stained using the Kluver‐Barrera protocol. This study decided to use sagittal sections instead of frontal sections due to the presumed course in which connections would run through the IA. Therefore, this would be more representative of any significant arrangements observed. The variation in staining protocols between the two studies is inconsequential. The Kluver–Barrera staining is a double stain protocol that combines CV staining for Nissl bodies with Luxol Fast Blue staining for myelin.^13^ The thalamus is a gray matter structure within the brain, and CV staining was deemed sufficient for the objectives of our study, which did not include identifying white matter within the IA and PVR regions. Despite using the Kluve–Barrera staining method in their study, Laslo and co‐workers^6^ did not report any findings regarding white matter/myelin within the IA and PVR.

Using light microscopy, this study observed no distinct neuronal structural arrangements in the IA or PVR regions. Furthermore, it should be noted that this study's findings do not concur with the findings of Laslo and co‐workers,^6^ and their results could not be replicated in either our study or any study within the known literature since 2005. Only five images, all of which appear to be of the same 55‐year‐old male specimen, were available in this publication,^6^ of which only a single circular arrangement is depicted. In addition, we do not observe any distinct circular arrangement of the neurons on the histological images, and this presumed pattern was not shown in the remaining five sample specimens for comparison in the article.

Malobabić and co‐authors^7^ reported four different types of neurons visible in the human IA, as observed with the Golgi‐Kopsch method: triangular neurons, fusiform neurons, multipolar neurons, and neurons with an oval perikaryon.^7^ However, in this study, pyramidal (triangular) neurons were observed, compared to their study, which mentioned that fusiform neurons are the most abundant type.

In contrast to our results and those of Laslo and co‐workers' study,^6^ recently published findings by Parra and co‐workers^14^ allude to the possibility of glial cell bridges within the IA but report no neuronal cell bodies in the same region.^14^ Therefore, they conclude that the IA is unlikely to be a gray commissure. However, their methodology included only H&E staining, which we have noted is not genuinely representative of observing neuronal cells within the tissue.^14^ We did, however, observe an abundance of glial cells and the presence of blood vessels in our sample, especially in the PVR region. Selcuk and Colakoglu^15^ also noted this when researching the most suitable staining method to distinguish between gray and white matter. They concluded that Kluver–Barrera staining, including CV staining, is one of the best protocols for observing neuroglia and Nissl bodies, whereas H&E is one of the best protocols for observing ependymal cells instead.^15^ This current study only used H&E and CV staining due to the availability of the reagents at the time of investigation.

Our study's design for this histological evaluation was purely observational. Therefore, no inferences can be made about the function and cytoarchitecture of the IA without further investigation. Although no specific organization of the cells could be found, our study has offered a novel view of the general histology of the thalamus, specifically of the IA and PVR, in midsagittal sections.

Unfortunately, vacuolation and damage to the cells in our sample were visible, but this did not negatively impact our observational findings. We did not use fresh brain tissue but rather embalmed cadaveric brains. The cell integrity and quality can be influenced by the fixation/embalming quality, which is dependent on several variables, including the fixation type, the degree of autolysis before fixation, the fixative solution and factors such as its constituents, temperature and osmolarity, as well as the fixative buffer used during the histological process.^16^ Also, the tissue can be damaged during physical processes, such as sectioning the tissues with a microtome or mishandling the specimens during the histological processes.

A thalamic commissure is said to exist within the IA, with fibers connecting the various thalamic nuclei, namely the midline, dorsomedial, paraventricular, ventral posteromedial, and intralaminar nuclei.^12^, ^17^, ^18^ Therefore, one would expect to find neurons within the IA and PVR regions. However, no other mention of a “thalamic commissure” was known to the authors in prior literature until recently. A significant finding was made in a probabilistic tractography study by Borghei and co‐authors.^10^ This study showed that the IA was vastly interconnected between the brain's limbic, frontal, and temporal lobes, insula, and pericalcarine cortices.^10^ The authors reported that the IA formed a stronger connectivity bridge in females than males.^10^ These findings may significantly alter the academic view of the IA in literature, lending it greater importance as a gray matter commissure, integral to some pathways within the brain, specifically to the amygdala, hippocampus, and entorhinal cortex.^10^ Another recent study by Chen and co‐workers^19^ reported findings of a robust thalamocortical signal circuit involving the IA. This circuit plays a role in cognitive function using perception and the transmission, processing, and integration of information.^19^

The future of IA studies should transition from the anatomical investigation to the functional investigation of this structure, as also emphasized by Sahin and co‐authors.^12^ This shift in focus in the literature will likely reveal insights into questions that have gone unanswered in prior literature. Although the IA's function is unknown, those without an IA do not appear to suffer any significant loss in neurological functions or significant differences from typical neurocognition. This is plausible due to fiber pathways through other commissures compensating for when there is no IA.^12^

## 5 CONCLUSION

This study confirmed the existence of neurons within the IA, forming a bridge between the PVR of the thalami. No specific structural organization of the neurons within the IA or PVR could be identified in this study with light microscopy. However, glial cells and blood vessels were identified. Therefore, a hypothetical link between the cytoarchitecture of the regions and their functions could not be analyzed. Even though the histological findings from this study differ from the known literature, this study proposed that the IA could serve as a potential bridge, or gray commissure, for neural pathways crossing over the brain's midline.

Further investigation of the function of the IA is crucial to understanding its role within the brain. Evidence of significant relationships between the IA and neuropsychological functions and behaviors will significantly influence the academic perception of this structure and the importance of brain commissures, consisting of white and gray matter fiber connections.

## 6 LIMITATIONS

The histological objectives of this study were updated due to unforeseen limitations in the original methodology. Due to the lack of observable neuronal organizational structures in the cytoarchitecture, we could not report on data such as the shape and appearance of neuronal organizational structures and the average size and frequency of neurons within a single organizational structure.

## AUTHOR CONTRIBUTIONS

Nicole van Heerden was the principal investigator responsible for the study design, data collection, experimental work, and manuscript drafting and revising. Lané Prigge and Gerda Venter provided supervision throughout the study, offering guidance on methodology, data interpretation, and critical revisions of the manuscript. All authors reviewed and approved the final version of the manuscript.

## CONFLICT OF INTEREST STATEMENT

The authors declare no conflicts of interest.

## ETHICS STATEMENT

Before commencing this study, the Research Ethics Committee of the Faculty of Health Sciences at the University of Pretoria approved it (Reference number: 3/2021). This study's ethical clearance pertains to undertaking an anatomically investigative study that does not include animals or living patients/volunteers and uses data collected from the brain specimens of donated bodies. These adult whole‐body donors, were bequeathed to the Department of Anatomy at the University of Pretoria in South Africa, where the brain dissections were also carried out. Anatomical studies of donated human bodies fall under the National Health Act, 61 of 2003 of South Africa and were completed following the ethical standards in the 1964 Declaration of Helsinki and all subsequent revisions.

## TRANSPARENCY STATEMENT

This study was conducted in adherence to the highest ethical and scientific standards. The research was designed, executed, and reported transparently, with all methods and results fully disclosed. All data, analytical methods, and materials used in the study are available upon request to ensure reproducibility and transparency. The authors confirm that there was no external influence on the study design, data collection, analysis, or interpretation of results.

## Data Availability

The data obtained and analyzed in this study can be made available from the corresponding author upon reasonable request.
